# Exploring the Detection of Cl^−^ Penetration in Portland Cement Mortars via Surface Electrical Resistivity

**DOI:** 10.3390/ma16227123

**Published:** 2023-11-10

**Authors:** Miguel Alberto Pablo-Calderón, Prisciliano Felipe de Jesús Cano-Barrita, Frank Manuel León-Martínez

**Affiliations:** CIIDIR Unidad Oaxaca, Instituto Politécnico Nacional, Calle Hornos No. 1003, Colonia Noche Buena, Sta. Cruz Xoxocotlán, Oaxaca 71230, Mexico; mpabloc1600@alumno.ipn.mx (M.A.P.-C.); fmleonm@ipn.mx (F.M.L.-M.)

**Keywords:** pozzolanic reaction, hydration, chloride diffusion, Friedel’s salt, pore solution

## Abstract

Surface electrical resistivity is a non-destructive technique that is sensitive to the microstructure of hydrated cement paste and the chemical composition of the pore solution in cement-based materials. In this study, a Wenner array was used to measure changes in mortar resistivity due to chloride ion diffusion as a function of electrode separation. Specimens were made from four mortar mixtures: 100% Ordinary Portland cement and 60% cement + 40% fly ash at two water/binder ratios of 0.55 and 0.40. The specimens were subjected to unidirectional chloride ion diffusion in a 2.8 M NaCl solution for 175 days. To determine the chloride penetration depth, three methods were used: silver nitrate spraying, chloride concentration profiles via potentiometric titration, and chloride concentration profiles via inversion of the resistivity data using the RES1D software (version 1.00.09 Beta). The results showed a linear relationship between the chloride ion penetration depth obtained via inversion of the surface electrical resistivity data versus the penetration depth from colorimetry and from chloride concentration profiling (both with R^2^ = 0.8612). Chloride penetration changed the conductivity of the pore solution; therefore, the resistivity decreased when increasing both the chloride concentration and the penetration depth. Inversion of surface resistivity data obtained with a Wenner array permitted non-destructive determination of chloride penetration. However, these results were obtained under laboratory environmental conditions and other scenarios must be addressed for wider applications.

## 1. Introduction

The durability of concrete structures is affected by chloride ion ingress, which can corrode the reinforcing steel bars. Chloride ions may enter cement-based materials through mixing with water or by external sources from environmental exposure [1]. Chlorides from external sources are mainly transported into the concrete through diffusion, which is influenced by factors like porosity, pore size distribution, ion exchange, and the chemical/physical binding of chlorides into the C-S-H and AFm phases. The capacity of chloride binding in hardened cement paste is affected by the C_3_A and C_4_AF content in cement, pore solution composition, addition of supplementary cementitious materials (SCMs), and leaching that alters the hydrate composition [2,3]. Chemical binding leads to the formation of Friedel’s salt, which removes chlorides from the pore solution [4]. The mechanisms of ingress and the binding of chlorides in cement systems are complex, as several processes are acting simultaneously. Porosity in cement-based materials is directly related to their mechanical properties and permeability. High porosity and a well-connected pore network facilitate the ingress of harmful species that cause the deterioration of reinforced concrete structures. Porosity and pore size distribution may be estimated using mercury intrusion porosimetry [5] or nuclear magnetic resonance relaxometry [6].

There are different standard techniques used to determine the ability of concrete to resist chloride ion ingress. One technique is the rapid chloride permeability test (RCPT) [7], which measures the amount of electrical charge passing through a saturated cylindrical concrete sample (100 mm in diameter and 50 mm in thickness) when a potential difference of 60 V of direct current is applied for six hours. This test has been subject to criticism, although it has been adopted as a standard and is widely used. The main drawbacks are that the current passed through the specimen is related to all ions in the pore solution; the measurements are made before steady-state migration is achieved; and the high voltage applied leads to an increase in temperature, which further increases the charge passed through. Another similar test is the non-steady-state migration test NT BUILD 492 [8]. This test applies an electrical potential to force chloride migration through the specimen, and the penetration depth is measured by spraying a silver nitrate solution on the fractured surface. A non-steady-state migration coefficient is then calculated. The AASHTO T 259 [9] is another type of test that measures the resistance of hardened concrete to chloride penetration by ponding the surface of concrete slabs with a three percent NaCl solution for 90 days. There is difficulty in interpreting the results because the specimens are subject to diffusion and wicking effects. Two additional tests, NT BUILD 443 [10] and ASTM C1556 [11], measure the accelerated penetration of chlorides in hardened concrete for the determination of the apparent diffusion coefficient and surface concentration based on chloride concentration profiles. These tests are expensive and time-consuming.

The techniques mentioned previously are destructive, which limits them to one measurement per test, and their results may show high variability [12]. On the other hand, there are non-destructive tests, such as ASTM C1876 [13] and AASHTO TP119 [14], that determine the bulk electrical resistivity or bulk conductivity of concrete. While these tests require molded specimens or specimens cored from actual structures, the surface electrical resistivity test on saturated concrete samples can indirectly assess the permeability of the material and provide an indication of its resistance to chloride ion penetration [15]. The Wenner array method is one test used for concrete electrical resistivity measurements that utilize four equally spaced surface electrodes. It is non-destructive [16], easy to use, rapid, and can be conducted on the same sample without special preparation [17]. According to Oleiwi et al. [18] and Cosoli et al. [16], the Wenner technique is one of the most widely used techniques for in situ tests on the surface of concrete. However, currently, there are still no widely accepted standards, other than AASHTO T358 [19], for measuring the surface electrical resistivity using the Wenner four-electrode method. Factors affecting resistivity measurements include porosity; pore size and connectivity; the tortuosity of the pore network and the composition of the pore solution, which are determined by the type of cementitious material; and the water/binder ratio (*w*/*b*). The curing method and sample conditioning also affect resistivity because they influence the properties of the pore solution, the degree of saturation, and temperature. Ambient temperature and relative humidity can be the most difficult conditions to control in real-life applications [20]. The accuracy of resistivity measurements is also dependent on the quality of the contact between the electrodes and the concrete surface. To create an electrolytic contact between the electrodes and the concrete surface, different solutions can be used. These include saturated sponges, soaked wooden plugs, a conductive gel, or localized wetting. However, these methods do not improve the contact between the electrodes and the concrete surface to a significant extent [20]. Studies indicate that the best resistivity measurements are obtained using the Wenner configuration with the largest electrode spacing [21]. An increase in separation will result in a greater penetration of the current field, covering more volume that results in lower relative values. In addition, resistivity measurements are substantially wrong if the semi-infinite geometry assumption is not met in small concrete specimens.

Regarding concrete resistivity and chloride penetration, there is an inverse correlation between resistivity and the chloride diffusion rate [22]. In addition, resistivity may be used to classify the risk of corrosion of a specific concrete structure, with low resistivities corresponding to a high risk of corrosion and vice versa. Qiao et al. [23] related the electrical resistivity of air-entrained concrete to the formation factor of the material. They found that increasing the air content decreased the formation factor of saturated concrete due to a higher volume of fluid-filled air voids. As w/c increased, the formation factor decreased due to a higher porosity and connectivity. Other uses of resistivity include the estimation of the chloride diffusion coefficient that may be required for the service life estimation of reinforced concrete structures [24]. Higher resistivities correspond to lower chloride diffusion coefficients. The only study identified in the literature related to the present study was that of Fares et al. [25], who inverted electrical resistivity tomography (ERT) data to obtain resistivity profiles as a function of depth. They used a Wenner configuration with 14 electrodes, two concrete mixtures, and a mortar exposed to chloride diffusion. The chloride profiles obtained via ERT were compared with those obtained using the chloride content of powder samples. They observed a good agreement between ERT and destructive testing.

The purpose of this study was to investigate the feasibility of determining the chloride ion penetration depth in mortar specimens under laboratory conditions by inverting surface resistivity data obtained with a commercial Wenner array instrument with variable electrode spacing. The resistivity measurements were performed as a function of time. The estimated chloride depth was compared with the penetration depth obtained from the colorimetric test using a 0.1 N AgNO_3_ solution sprayed on a freshly broken surface, as well as from chloride content profiles at a depth where the concentration was approximately zero. The relationship between the depths obtained non-destructively and those obtained using standard destructive tests will be explored.

## 2. Materials and Method

### 2.1. Materials

**Figure 1:**
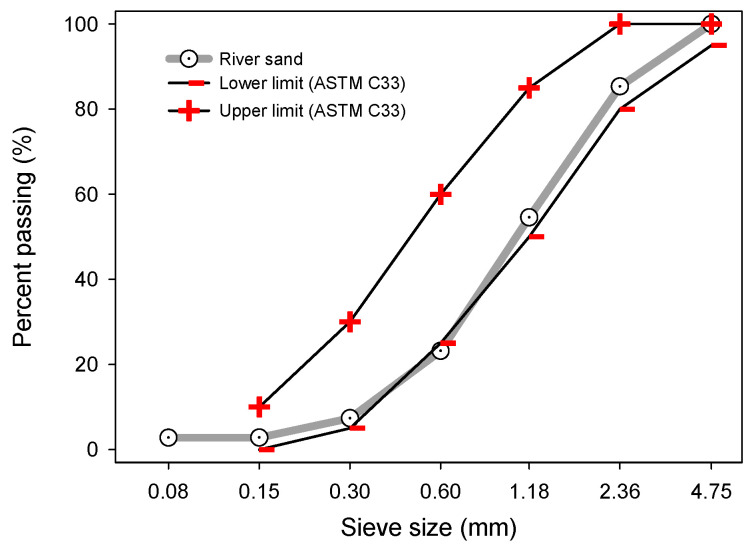
River sand grading used to prepare the mortar mixes.

Ordinary Portland cement (OPC), class F fly ash (FA), and river sand were used. The relative density of the sand was 2.66 g/cm^3^, with an absorption of 2.81% [26] and a fineness modulus of 3.28. The chemical composition of the cementitious materials used is shown in Table 1. The river sand grading is provided in Figure 1.

### 2.2. Method

#### 2.2.1. Preparation and Conditioning of Mortar Specimens

**Figure 2:**
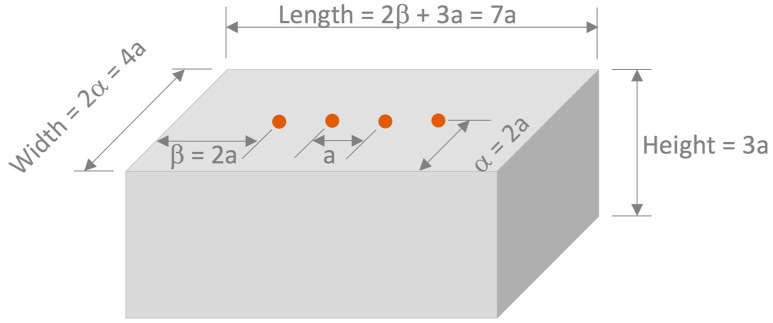
Criteria for estimating minimum specimen dimensions to minimize edge effects in surface electrical resistivity (r) measurements (geometric correction factor of approximately 1, and *a* represents the electrode spacing) [21,31].

Mortar mixes with water/binder (*w*/*b*) ratios of 0.55 and 0.40 and a sand/binder (*s*/*b*) ratio of 2.75 were prepared according to the procedure described in the ASTM C192 standard [28]. Table 2 provides the mortar mixture proportions. The sand/binder ratio (*s*/*b*) of 2.75 was used as proposed in ASTM C109 [29] for preparing the Portland cement mortars. The *w*/*b* ratios were selected as 0.55 and 0.40 in order to produce relatively high and low chloride ion diffusion coefficients and low and high electrical resistivities. Two mortars used 100% OPC and the other two used 60% OPC + 40% FA, by mass, which were labeled as OPC0.55, FA0.55, OPC0.40, and FA0.40, respectively. FA was included in the mixes because it significantly reduces the porosity and pore size distribution in hardened cement paste, which, in turn, reduces the electrical resistivity of concrete. In addition, cement pastes containing FA chemically bind a higher amount of chlorides compared with plain OPC cement pastes. A total of 18 mortar specimens were cast from each mortar mix. Table 3 provides details on the shape and dimensions of the different specimens. As is shown in Figure 2, the size of the prismatic mortar specimens was determined to minimize edge effects and to consider the space as semi-infinite, taking the value of *a* as less than 1/3 of the thickness of the specimens (approximately *a* = h/3) [21,30,31]. Parameters α and β are dependent on the electrode spacing. The former represents the distance from the center of the electrode to the edge of the prism and it is perpendicular to the electrode array; β is the distance from the external electrode to the edge of the prism and it is parallel to the electrode array. Therefore, the dimensions were 25 cm in width, 40 cm in length, and 15 cm in height. A geometric correction factor of approximately 1.0 was obtained from the specimen size to minimize edge effects in the surface electrical resistivity measurements [21,31].

The specimens were cured via immersion in a calcium-hydroxide-saturated solution for 28 and 220 days. The longest curing time was chosen to reduce the effect of hydration reactions on the resistivity measurements. Control specimens remained in the curing conditions throughout the experiment. After the curing period, a marine epoxy coating was applied to the surface of the prismatic and cubic specimens, leaving one face of the prisms (25 cm × 40 cm) and one face of the cubes without any epoxy coating. By leaving the epoxy-coating-free faces in a 2.8 M sodium chloride solution, according to the NT BUILD 443 standard [10], the specimens were exposed to unidirectional chloride ion diffusion for 7, 28, 112, and 175 days before testing. The control specimens were kept in a saturated calcium hydroxide solution. As an accelerated test, NT BUILD 443, requires a NaCl concentration that is approximately 5 times the concentration found in seawater.

#### 2.2.2. Surface Electrical Resistivity Measurements

**Figure 3:**
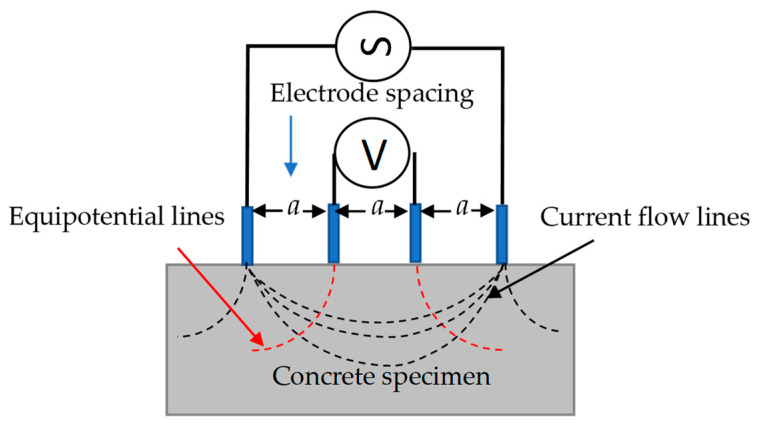
Illustration of the four-point Wenner array to measure surface electrical resistivity.

**Figure 4:**
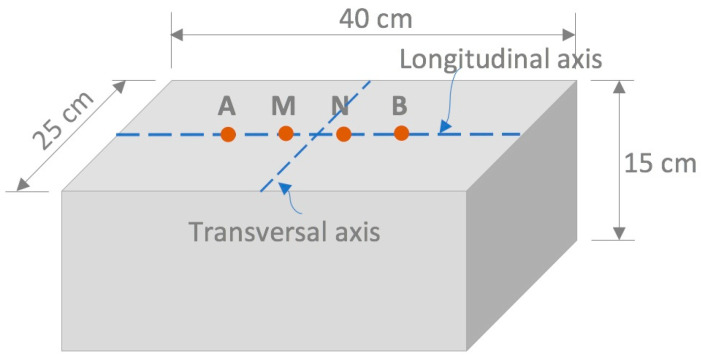
Diagram of the location of the reference axes used in electrical resistivity measurements as a function of electrode separation *a.* A, M, N, and B are the electrode positions in the specimen.

The surface electrical resistivity was measured using a Resipod resistivity meter (Proceq, Schwerzenbach, Zurich, Switzerland) with a Wenner array and a geometric fixture with variable electrode separation (Figure 3). There are four electrodes in the Wenner array: two external and two internal. External electrodes (A and B) apply an alternating current, while the internal electrodes (M and N) measure the potential difference. To avoid edge effects and, consequently, an overestimation of the resistivity values, the prism size was calculated according to the recommendations provided by Gowers and Millard [31] and Chun-Tao Chen et al. [32]. The resistivity readings were taken at the center along the longitudinal direction of the sample. Two axes perpendicular to each other, one longitudinal and the other transverse, were sketched on the surface of the specimens (Figure 4). The separations between the electrodes *a* were 3.8, 4.0, 4.2, 4.4, and 4.6 cm. The resistivity tests were performed on the saturated, surface-dry specimens under laboratory conditions at approximately 23 ± 1 °C. Four test groups were studied, two of which were prismatic specimens with 28 and 220 days of curing and were exposed to different chloride diffusion times. The third group had chloride-free prismatic and cylindrical specimens with 28 days of curing and *a* = 38 mm. Finally, the fourth had prismatic and cylindrical specimens with 220 days of moist curing and were exposed to different chloride diffusion times and using a value of *a* = 38 mm.

Three readings were taken for each electrode separation, and the mean and standard deviation were obtained. The electrical resistivity was calculated according to Equation (1), assuming a semi-infinite, homogeneous, and isotropic volume [13,14]:

*ρ* = 2π*aR* (1)

where *R* is the electrical resistance, *ρ* is the apparent electrical resistivity, and *a* is the electrode separation of the Wenner array.

AASHTO T358 [19] requires correction of the resistance values based on curing in a saturated calcium hydroxide solution, which was carried out using Equation (2):

*ρ_corrected_* = *ρ k_LW_* (2)

where *ρ _corrected_* is the true resistivity and *k_LW_* is a correction factor for curing the sample in a saturated calcium hydroxide solution (*k_LW_* = 1.1).

#### 2.2.3. Data Inversion Using the RES1D Free Software

Using the RES1D free software [33], the thickness of two layers with different electrical properties (with and without chlorides) was determined. The program read a data file that contained information such as the estimate of the thickness of the surface layer, at least five data pairs of the electrode separation and their corresponding apparent resistivities (*a*, *ρ*), the number of layers sought, their resistivity (*ρ*), and their thickness (z). To reduce the difference between the observed and calculated values, the model and optimization subroutine modified layer thickness and resistivity were compared. The result of the data inversion represented the depth of the two layers described above.

The initial first layer thickness (penetration depth) values required for the inversion process were estimated with the solution according to Fick’s second law of diffusion. The penetration depths were calculated at approximately a 0.0% chloride concentration, assuming a surface concentration of C_s_ = 1%. The diffusion coefficients were obtained using Equations (3)–(6) [34,35,36] based on a reference diffusion coefficient that considers the type of binder and *w*/*b* ratio:

*D(t)* = *D_ref_ (t_ref_/t)^m^* (3)

where *D(t)* is the diffusion coefficient (m^2^/s) at time *t*; *D_ref_* is the diffusion coefficient (m^2^/s) at reference time *t_ref_*; and *m* is a constant that depends on the type and amount of binder [37].

*D*_28_ = 10^(−12.06+2.4*w*/*b*)^ (4)

m = 2.5 (*w*/*b*)^−0.6^ (5)

m = 0.2 + 0.4 (% FA/50)(6)

where *D*_28_ is the reference diffusion coefficient (m^2^/s); *w*/*b* is the water/binder ratio; and % FA is the percentage substitution of Portland cement with fly ash in the mix [35,38].

According to Polder et al. [39] and Cosoli et al. [16], the thickness of the second layer was *z* ≈ *a* because the resistivity measured with the Wenner configuration is considered as the average of a hemisphere of radius equal to the electrode spacing *a*, as long as the hypothesis that the medium is homogeneous and semi-infinite is valid.

#### 2.2.4. Chloride Penetration Depth Using the Colorimetric Technique

The specimens were fractured along their longitudinal axis, which coincided with the axis of the electrical resistivity measurements. To conduct the colorimetric test, a freshly fractured sample was sprayed with a 0.1 N silver nitrate (AgNO_3_) solution [40]. Silver chloride precipitated, creating a whitish region due to the rapid chemical reaction between the free chlorides and silver nitrate. This color change shows the depth of chloride penetration. The color change prevailed with time, and it was observed that the depth increased if it was measured several hours later. For this reason, measurements were taken every 10 mm with a Vernier caliper, first after spraying AgNO_3_ and then 24 h later.

#### 2.2.5. Chloride Content Profiles

Once the specimens used for the colorimetric tests were fractured at 7, 28, 112, and 175 days, a PF-1100 profile grinder (Germann Instruments, Evanston, IL, USA) was used to extract powder samples in 2 mm thick layers (5–7 g per layer). Finally, the powder was processed to determine the acid-soluble chloride ion content via potentiometric titration.

## 3. Results and Discussion

### 3.1. Electrical Resistivity Measurements

**Figure 5:**
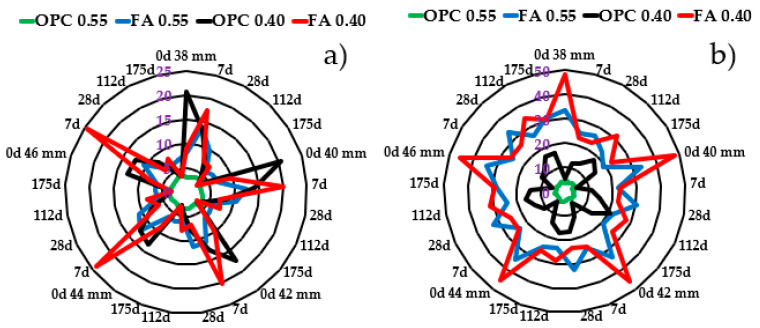
Electrical resistivity in mortar specimens with (**a**) 28 and (**b**) 220 days of moist curing at different Cl^−^ exposure times as a function of electrode spacing: 38, 40, 42, 44, and 46 mm.

Figure 5 presents the electrical resistivity data versus electrode spacing in radar-type graphs for specimens that underwent (a) 28 and (b) 220 days of moist curing. The graphs show variations in the electrical resistivity of the specimens as Cl^−^ diffused, which resulted from the exposure time, electrode separation, *w*/*b* ratio, and the type of cementitious materials. In all cases, the percentage variations in resistivity with respect to the zero day of chloride exposure (considered as the control) were calculated.

When the separation of electrodes was 38 mm, it was observed that the resistivity of OPC0.55 at zero days was the lowest (3.1 kΩ·cm) compared with the other three mortars; although, by test day 175, it increased by approximately 39% of its initial value. A different behavior was exhibited by the OPC0.40, which showed the highest initial resistivity of 20.8 kΩ·cm and decreased by 35.4% at seven days. At 175 days, the resistivity decreased by 85.9%. For the FA0.55 mortar, the resistivity started at 8 kΩ·cm, increased by 73% at seven days, and then gradually decreased until it reached 18% below its initial value at 175 days. A similar behavior was exhibited by the FA0.40, which started at 9.1 kΩ·cm and increased by 91% at seven days, and then gradually decreased until it reached 72% below its initial value. Increasing the electrode separation to 40 mm caused the resistivity to decrease by approximately 5%.

A similar behavior between zero and seven days has been described by Wang et al. [41], who observed that the resistivity does not decrease instantaneously with the ingress of Cl^−^ because there is no contribution to the electric current flow at the moment of chloride ion binding to the hydration products. Similarly, Loche et al. [42] reported that Cl^−^ ions modified the electrical response of cement-based materials. As the Cl^−^ ions penetrated, the electrical resistance initially increased and then decreased, which is in agreement with the behavior of specimens FA0.55 and FA0.40, which were moist-cured for 28 days. As electrical resistivity depends on the chemical composition of the pore solution, the presence of other ions, such as K^+^, Na^+^, OH^−^, and, to a lesser extent, Ca^2+^ and SO^2−^, could affect the resistivity measurements [43]. On the other hand, Minagawa et al. [30] found that electric current flow increases in the region of lower electrical resistivity, and Oleiwi et al. [18] observed that as the Cl^−^ content in the pore solution increases, the resistivity decreases. The latter is in agreement with the findings of Polder et al. [22] and Cosoli et al. [16], who consider the decrease in resistivity to be related to the presence of chloride ions.

Increased electrode spacing allowed deeper probing into the sample [21,31]; for example, by changing *a* from 38 mm to 46 mm, the fraction of the analyzed volume that had electrical properties of the “layer” containing chloride ions decreased and the measurements better reflected the properties of the zone that was free from these ions. Using this strategy, the current flow in the mortar is more homogeneous and the variability in the measurements is lower [22]. The resistivity also decreased as the *w*/*b* ratio increased, as expected, because of a larger porosity and connectivity.

In contrast to the mortar specimens that were moist-cured for 28 days, where the benefits of using fly ash were marginal, the resistivity of mortars with long moist curing showed a completely different behavior. Figure 5b shows that sample OPC0.55 had the lowest resistivity, with similar values and behavior to the sample that was moist-cured for 28 days. The resistivity started at 3.4 kΩ·cm and remained nearly constant during the chloride exposure time (175 days). Sample OPC0.40 showed the effects of a lower *w*/*b* ratio. Its resistivity started at 5.6 kΩ·cm and increased 2.16 times at seven days, then gradually increased until reaching 2.64 times its initial value at 175 days. For sample FA0.55, the initial resistivity value was much higher (33.8 kΩ·cm) than samples containing only cement. Its resistivity decreased by 26% in seven days and continued decreasing by up to 44% of its initial value. A similar behavior was observed for sample FA0.40, which started at a resistivity of 48.3 kΩ·cm and decreased by 53% at seven days; then, it increased at 112 days and decreased again at a resistivity value similar to the one measured at seven days. Increasing the electrode separation (40–46 mm) caused a decrease in the resistivity (by approximately 9%). There was also a decrease in resistivity as the *w*/*b* ratio increased, as expected.

Specimens that were moist-cured for 28 days had a lower surface resistivity than those cured for 220 days due to the significant cement hydration effects at the early stages, with resistivities ranging from 3 to 20 kΩ⸱cm. In contrast, at the longest curing time, the resistivity ranged from 3 to approximately 50 kΩ⸱cm (*ρ* OPC0.55 < *ρ* OPC0.40 < *ρ* FA0.55 < *ρ* FA0.40) due to the denser microstructure and higher degree of hydration. Specimens with a higher *w*/*b* ratio showed lower resistivity than those with a lower *w*/*b* ratio. Moreover, specimens with FA had higher resistivity than those constructed only with OPC. This is because of the pozzolanic reaction of FA that produced more C-S-H, thus decreasing the porosity and refining the pore structure. The formation of solid phases such as Friedel’s salt may affect the resistivity.

#### 3.1.1. Electrical Resistivity of Chloride-Free Specimens

**Figure 6:**
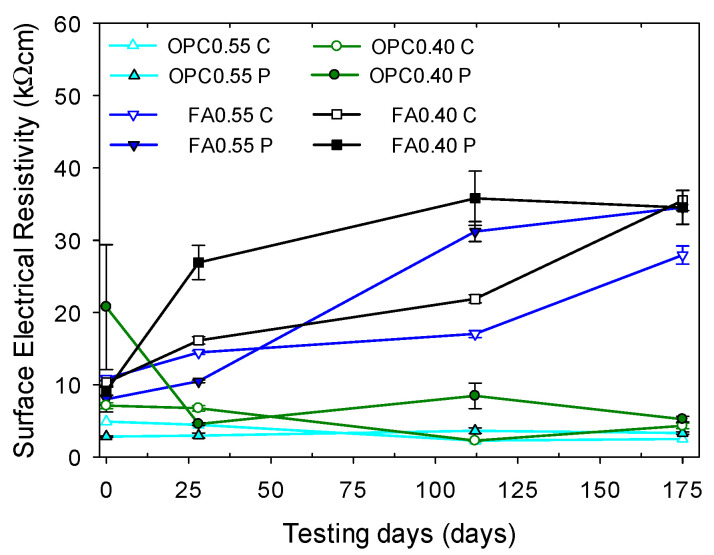
Evolution of electrical resistivity (using *a* = 38 mm) in control prismatic and cylindrical specimens after 28 days of moist curing. Error bars represent ± one standard deviation.

Figure 6 shows the resistivity evolution as a function of age, *w*/*b* ratio, type of cementitious materials, and specimen geometry. Measurements were performed at 0, 28, 112, and 175 days. The age of the specimens was obtained from the sum of the 28 days of moist curing plus the testing day. As expected, due to hydration reactions, resistivity increased with age in most of the specimens, especially in those containing FA. According to Grazia et al. [44], FA and other SCMs impact concrete’s microstructure and pore solution chemistry (alkalinity reduction). They found that the resistivity may increase by up to ten times when adding SCM due to the formation of additional C-S-H, which acts as an electrical barrier. The pozzolanic reaction leads to the partial consumption of portlandite from the system and a decrease in pH. According to Medeiros and Lima [45] and Kang et al. [46], blended cements have higher resistivity than OPC. Although class F FA slows down the C-S-H formation, in the present study, the resistivity was still improved compared with OPC mortars. Reducing the *w*/*b* ratio from 0.55 to 0.40 also causes an increase in resistivity due to a reduced porosity and connectivity of lower *w*/*b* ratio mortars. Hou [47] observed that the microstructure of the C(A)SH gel conducts water molecules and ions less easily due to the addition of Al^3+^.

The shape and size of the specimens had a significant influence on the resistivity, and in all cases, cylindrical specimens showed lower values than prismatic ones. The dispersion of the measured resistivity, expressed by the standard deviation bars, was smaller for cylinders than for prisms due to the propagation geometry of electric waves [48,49]. This is in agreement with the investigations of Hornáková and Lehner [50], who determined that the variance in measured resistivity values in concrete specimens, shown through standard deviation, was two to three times lower for cylindrical geometry than for prismatic geometry.

#### 3.1.2. Electrical Resistivity of Mortars without Hydration Effects Exposed to Chlorides

**Figure 7:**
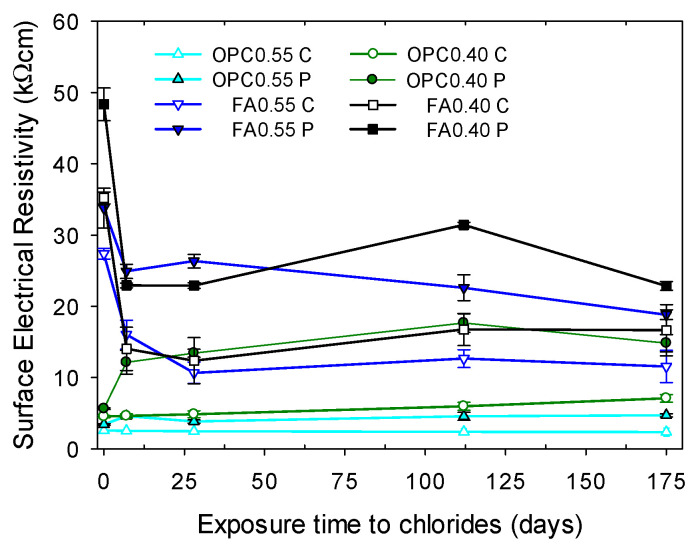
Electrical resistivity of cylindrical (C) and prismatic (P) specimens as a function of exposure time to a 2.8 M NaCl solution, using *a* = 38 mm. The specimens were previously moist-cured in a saturated calcium hydroxide solution for 220 days. Error bars represent ± one standard deviation.

Figure 7 compares the resistivity of cylindrical and prismatic specimens after 220 days of moist curing that were then subjected to chloride ion diffusion. The effect of hydration on the resistivity of these specimens was expected to be negligible because of the long moist-curing time. As chloride ions diffused into the samples, the resistivities exhibited a distinct behavior depending on the mix composition (mortars containing FA or only OPC), type of specimen, and *w*/*b* ratio. Mortars containing only OPC exhibited the lowest resistivities, as mentioned in Section 3.1. The resistivity of the OPC0.55 specimens remained nearly constant during the 175 days. Specimens of OPC0.40 mortar with prismatic geometry demonstrated a twofold increase at seven days; then, the resistivities remained nearly constant. Contrary to this behavior, the cylinders showed a constant resistivity up to 28 days, and then it slightly increased at 112 and 175 days.

Mortars containing FA exhibited a different behavior. After seven days, the resistivity of the FA0.55 specimens decreased by around 50% and then continued to decrease gradually. The resistivity of the FA0.40 specimens also significantly decreased at seven days (by approximately 53%), but then it remained nearly constant or slightly increased at 112 and 175 days. As was shown in the previous section (Section 3.1.1), cylindrical specimens had a lower resistivity than prismatic specimens.

### 3.2. Chloride Penetration Depth Using Three Different Techniques

**Figure 8:**
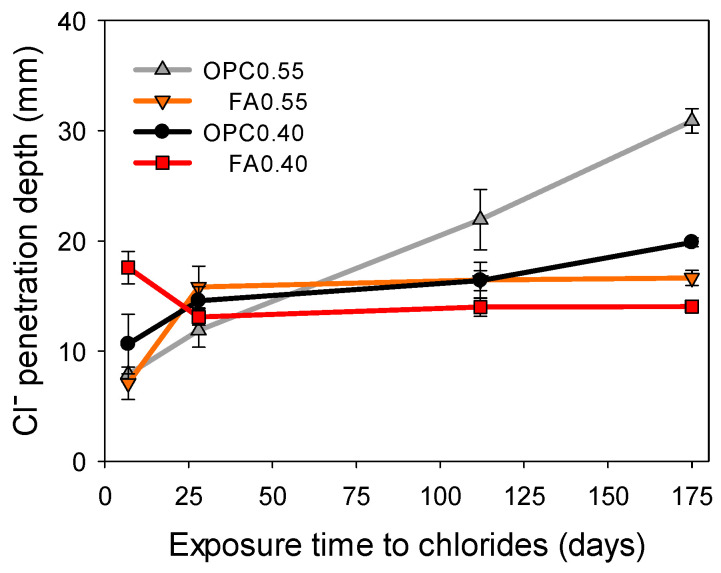
Chloride penetration depth versus the exposure time from AgNO_3_ solution spraying.

Figure 8 illustrates the chloride penetration depth measured in the mortar specimens immediately after spraying silver chloride on the fractured surface. As expected, the penetration depth increased with chloride exposure time. At the end of the exposure to chlorides (175 days), the highest penetration was observed for the OPC0.55 mortar, followed by OPC0.40. The lowest penetration depth was seen for FA0.55, and FA0.40 had the highest resistivity. The chloride concentration for color change depends on the amount of OH^−^ ions in the pore solution. This ranges from 0.28 to 1.69% by mass of cement or between 0.072 and 0.714 mol/L for the AgNO_3_ technique [51]. In addition, there is no consensus on the chloride concentration corresponding to the color change boundary. The average level of soluble Cl^−^ detected at the color change boundary was 0.9 wt.% cementitious materials or 0.12 wt.% concrete, with high coefficients of variation of 33% and 40%, respectively [40]. The amount of AgCl and Ag_2_O formed at the color change boundary depends on the Cl^−^/OH^−^ ratio in the pore solution and can be seen as an estimation of the chloride ion penetration depth, rather than the actual boundary between chloride-containing and chloride-free areas.

**Figure 9:**
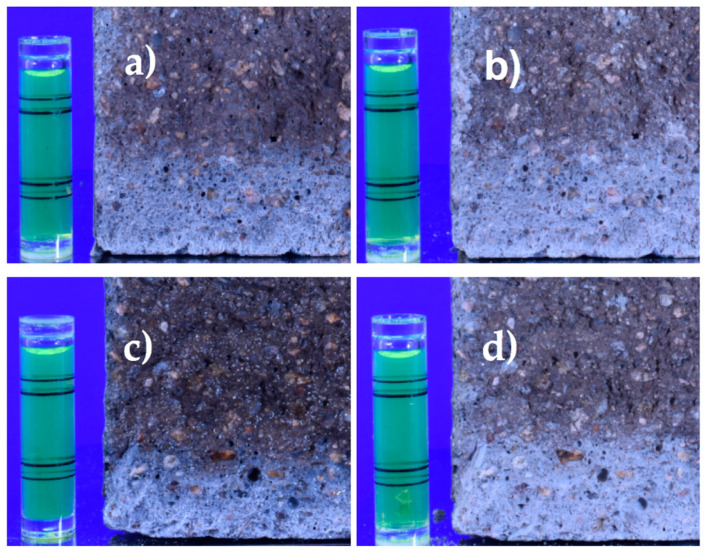
Change in penetration depth with time in the colorimetric test: (**a**) OPC0.55 at 0 h, (**b**) OPC0.55 at 24 h, (**c**) FA0.55 at 0 h, and (**d**) FA0.55 at 24 h after spraying the AgNO_3_ on the broken surface. The bubble level was 40 mm in height.

When performing the colorimetric test, the presence of a diffuse zone was detected between the zone with and without chlorides, which was difficult to measure immediately after spraying with AgNO_3_. Therefore, the decision was made to keep the samples under the laboratory conditions, and a second measurement was performed 24 h later. The second measurement showed a higher chloride penetration depth than that observed immediately after spraying. Figure 9 shows photographs from two specimens at 0 and 24 h after spraying. The white region depth increased by an average of 27% at 24 h after spraying the AgNO_3_ solution. To the best of our knowledge, this observation has not been reported in the existing literature.

SCMs change how the hydrated cement paste phases are formed, and thus, their Cl^−^ binding properties [52]. For example, FA is a material rich in reactive aluminates and exhibits increased AFm formation and, therefore, increased Cl^−^ binding in the C(A)SH. A decrease in the amount of Ca^2+^ causes a decrease in the amount of Cl^−^ bound to AFm and an increase in the amount bound to C(A)SH since it is a portlandite-deficient system. The ingress of Cl^−^ reduces the adsorption of Al^3+^ from C(A)SH and increases the amount of Al^3+^ available to form chloride-bound AFm.

In the present study, a 2.8 M NaCl concentration was used; therefore, the Cl^−^ ions entering the system must compete to displace the SO_4_^2−^, OH^−^, and/or CO_3_^2−^ anions of the different monosulfoaluminates and, finally, Friedel salt is formed [4]. According to Balonis et al. [4], at high chloride concentrations the Kuzel salt or monocarboaluminate phases are destabilized by increasing the chemically bound chloride so that a high chloride concentration would make the formation of the AFm phases, such as the Kuzel salt, impossible. When the systems destabilize, ions such as sulfate ions can be released, and they react with calcium and aluminum, forming ettringite and causing expansion due to the increase in the molar volume of the solid and refining of the porosity [4].

**Figure 10:**
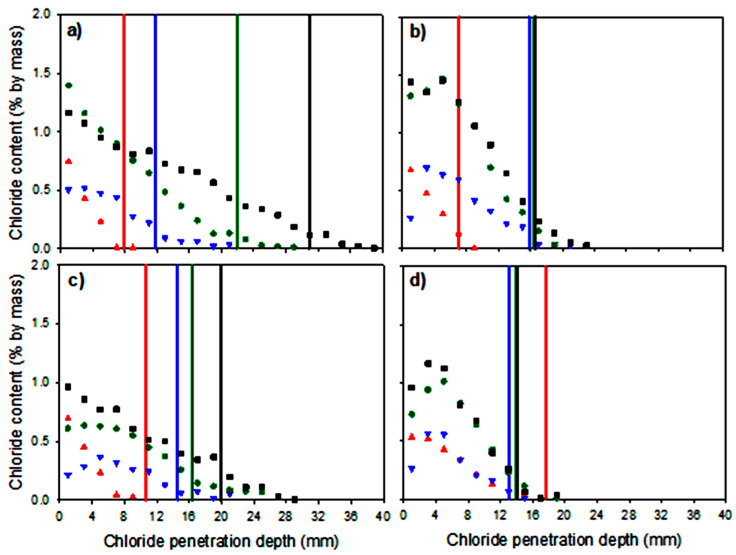
Total chloride content profiles and chloride penetration depth determined using colorimetry after spraying and at 7 (red), 28 (blue), 112 (green), and 175 (black) days of exposure to chlorides: (**a**) OPC0.55, (**b**) FA0.55, (**c**) OPC0.40, and (**d**) FA0.40.

Figure 10 shows the chloride content profiles for the mortar specimens obtained via potentiometric titration, as well as the chloride penetration depth obtained by spraying the AgNO_3_ solution. Figure 10a shows that OPC0.55 had the highest Cl^−^ penetration, reaching a depth of approximately 38 mm at 175 days of chloride diffusion, whereas for FA0.55, the depth reached 22 mm (Figure 10b). The latter was expected because of the presence of FA. In the same manner, OPC0.40 (Figure 10c) had a 26 mm penetration, and FA0.40 (Figure 10d) exhibited the lowest penetration (18 mm). The measurements were taken at the color change boundary immediately after spraying the freshly fractured surfaces with the AgNO^3^ solution. This test underestimates the chloride penetration depth.

Table 4 presents the apparent diffusion coefficients calculated by fitting the data of the chloride content profiles shown in Figure 10 to the solution of Fick’s second law of diffusion [34]. The trends observed in the diffusion coefficient from 28 days up to 175 days of exposure to chlorides show higher values for the high *w*/*b* ratios and lower values for the low *w*/*b* ratios, as expected. Moreover, the mortars containing fly ash exhibited lower diffusion coefficients than the mortars containing only OPC because of the pozzolanic reaction that produces additional C-S-H and reduces porosity and permeability.

**Figure 11:**
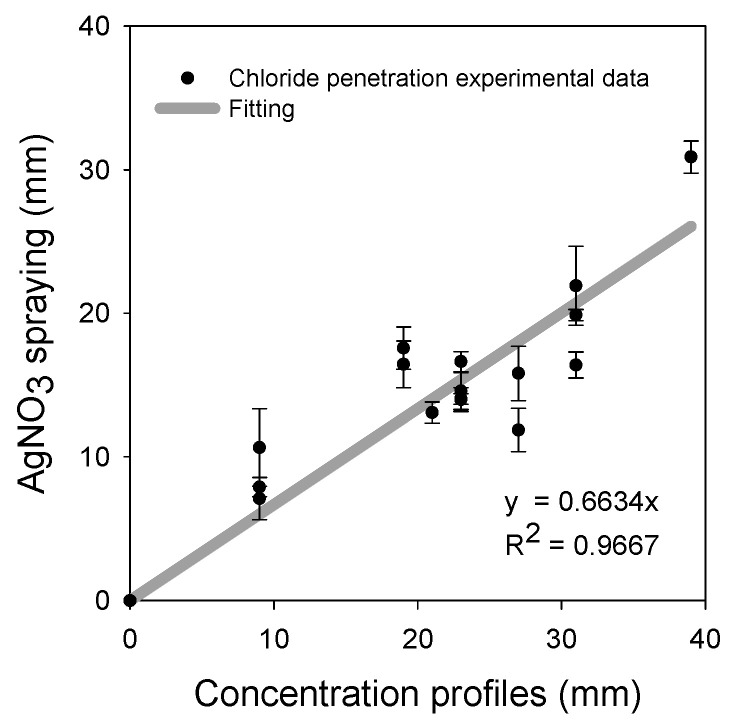
Relationship between the penetration depth of Cl^−^ in mortar specimens at different days of chloride exposure obtained using colorimetry after spraying a 0.1 N AgNO_3_ solution, and the distance from concentration profiles. Error bars represent ± one standard deviation.

A linear relationship between the penetration depths from spraying the AgNO_3_ solution and the concentration profiles is shown in Figure 11. There is a linear correlation (R^2^ = 0.96) between them with a slope < 1, indicating an underestimation with the colorimetric method.

**Figure 12:**
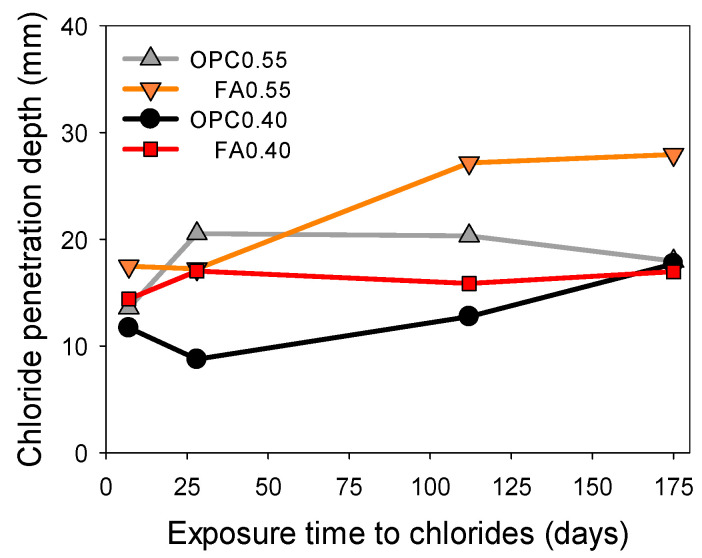
Chloride penetration depth in mortar specimens OPC0.55, FA0.55, OPC0.40, and FA0.40 obtained via inversion of resistivity data at different exposure times to 2.8 M NaCl solution.

Another alternative way of obtaining the depth of chloride penetration proposed in this study is by inverting the surface electrical resistivity data obtained at different exposure times to 2.8 M NaCl. Figure 12 shows that, as expected, for mortars with high *w*/*b* ratios or the case of fly-ash-free mixtures, the chloride penetration was higher.

**Figure 13:**
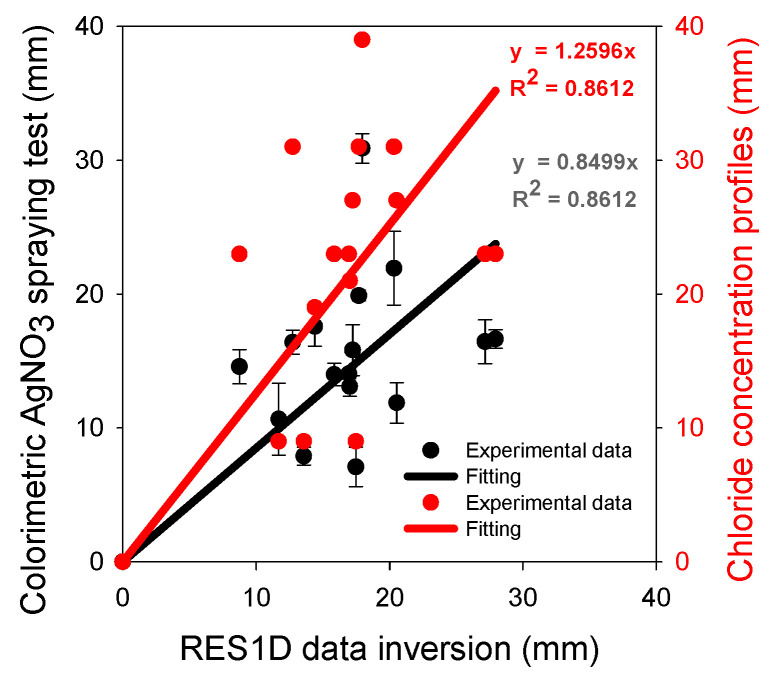
Correlation between Cl^−^ penetration depth obtained via resistivity data inversion and that obtained using the colorimetric test and chloride content profiles on mortar specimens on different days of chloride exposure. Error bars represent ± one standard deviation.

Figure 13 shows two linear correlations of the chloride penetration depth determined through inversion of the resistivity data at different Cl^−^ exposure times. The first of these is R^2^ = 0.86, determined by comparing it with the depth of chloride ion penetration obtained via the colorimetric method, while the second is R^2^ = 0.86, determined by comparing it with the depth of chloride ion penetration determined via the chloride content profiles. The first correlation indicates that the resistivity measurements are underestimates and the second one overestimates the chloride penetration. Fares et al. [25] obtained chloride profiles using electrical resistivity tomography (ERT) and compared them with those obtained using the chloride content of powder samples. They observed a good agreement between ERT and the destructive test because they used a smaller electrode separation than in our study, which was restricted to a minimum of 38 mm. In addition, their electrodes maintained a fixed relative position to perform the measurement with all electrode separations, which resulted in a lower variability of measurements. 

## 4. Conclusions

This study explored the use of a simple Wenner array to measure changes in the surface electrical resistivity of mortars subject to chloride diffusion under laboratory conditions as a function of electrode separation. The following conclusion is given: Diffusion of chlorides in mortar specimens affects the resistivity measured with a simple Wenner array with variable electrode separation after inversion with the RES1D software, which permits indirect calculation of the chloride penetration depth. This was validated through a linear correlation with AgNO_3_ spraying and chloride concentration profiling, both resulting in an R^2^ of approximately 0.86.

## Figures and Tables

**Table 1:** Chemical composition (% by mass) of the cementitious materials used [27].

Main Oxides	SiO_2_	Al_2_O_3_	Fe_2_O_3_	CaO	TiO_2_	P_2_O_5_	MgO	Na_2_O	K_2_O	SO_3_	LOI
OPC	21.07	3.69	4.50	61.93	0.97	0.10	1.83	0.09	0.30	2.54	4.38
FA	62.28	20.38	4.09	4.68	0.94	0.38	0.98	0.31	0.99	-	3.43

**Table 2:** Mortar mixture proportions.

Material	Mix			
(kg)	OPC0.55	FA0.55	OPC0.40	FA0.40
Cement	518	301	562	326
Fly ash	-	200	-	217
Sand	1425	1381	1544	1493
Water	285	276	225	217

**Table 3:** Characteristics of mortar specimens prepared from each mix.

Test	Type	Dimensions				Quantity
		w (cm)	l (cm)	h (cm)	Ø (cm)	
Surface electrical resistivity	Control prisms	25	40	15		2
	* Prisms	25	40	15		2
	Control cylinders		20		10	4
Colorimetry/chloride content profiles	Control cubes * Cubes	15	15	15		2
		15	15	15		8

* Specimens for measurements at 7, 28, 112, and 175 days of exposure to chloride ion diffusion.

**Table 4:** The apparent diffusion coefficient and surface chloride concentration of mortar specimens moist-cured for 28 days determined by fitting chloride content profiles to the solution of Fick’s second law of diffusion [34].

Exposure Time	OPC0.55		FA0.55		OPC0.40		FA0.40	
	D	C_s_	D	C_s_	D	C_s_	D	C_s_
(Days)	(m^2^/s)	(%)	(m^2^/s)	(%)	(m^2^/s)	(%)	(m^2^/s)	(%)
7	2.6 × 10^−11^	1.09	4.1 × 10^−11^	0.98	3.3 × 10^−11^	1.00	1.2 × 10^−10^	1.85
28	6.8 × 10^−11^	0.79	5.0 × 10^−11^	1.08	7.0 × 10^−11^	0.70	2.9 × 10^−11^	0.96
112	1.4 × 10^−11^	1.62	5.6 × 10^−12^	3.00	1.8 × 10^−11^	1.04	5.0 × 10^−12^	2.12
175	2.6 × 10^−11^	1.41	5.6 × 10^−12^	2.46	1.4 × 10^−11^	1.00	4.0 × 10^−12^	1.85

## Data Availability

The data presented in this study are available on request from the corresponding author.
